# Histopathological Changes of the Corticospinal Tract Following Hemorrhagic and Ischemic Stroke

**DOI:** 10.3390/brainsci15080864

**Published:** 2025-08-13

**Authors:** Sarolta Kollai, Dániel Bereczki, Balázs Dobi, Tibor Glasz, Tibor Kovács

**Affiliations:** 1 Department of Neurology, Semmelweis University, Balassa u. 6, 1083 Budapest, Hungary; kollai.sarolta@semmelweis.hu (S.K.); bereczki.daniel@semmelweis.hu (D.B.); 2 Károly Schaffer Laboratory of Neuropathology, Department of Neurology, Semmelweis University, 1083 Budapest, Hungary; 3 HUN-RENSU Neuroepidemiological Research Group, 1083 Budapest, Hungary; balazs.dobi@gmail.com; 4 Department of Pathology, Forensic and Insurance Medicine, Semmelweis University, 1091 Budapest, Hungary; glasz.tibor@semmelweis.hu

**Keywords:** Wallerian degeneration, medulla oblongata, histology, cerebral infarct, cerebral hemorrhage

## Abstract

Previous data on the time course of corticospinal tract (CST) degeneration after stroke are scarce, especially in the early phase. Using post-mortem histomorphological and immunohistochemical methods, CST degeneration (its temporal dynamics and its association with stroke type) was investigated in the medulla oblongata pyramids in twenty-two cases: nine patients with ischemic stroke and eleven with hemorrhagic stroke and two patients with very long survival after multiple strokes. CD68 and neurofilament immunohistochemistry and Klüver–Barrera staining were applied to assess microglial activation and axonal and myelin degeneration, respectively, and statistical methods were used to investigate the relationship between microglial density, stroke type, and survival time. Strong microglial activation starts on day 3 after stroke and increases over time, with the first signs of axon degeneration and myelin degradation observed from day 7 after stroke. Histological changes and their temporal dynamics do not differ between hemorrhagic and ischemic stroke. Our results show that myelin degeneration starts earlier than previously suggested and is observed almost simultaneously with axon degeneration, whereas microglial activation increases steadily from day 3 onwards in both ischemic and hemorrhagic stroke.

## 1. Introduction

Wallerian degeneration (WD), the programmed, stereotyped degeneration of the axon due to nerve cell injury, was first observed and described on a peripheral nerve by Waller more than 150 years ago [1,2]. The original belief—lasting for a century and a half—that nutritional failure leads to degeneration was disproved with the discovery of the so-called slowWD (WldS) mice. This is a strain of mice in which WD is significantly delayed compared to the wild type. It was concluded that WD is an active, programmed process, similar to apoptosis [3,4]. Its mechanism is incompletely understood but has a set of time-dependent molecular, cellular, and morphological characteristics [1,3]. Since then, it has been well established that in addition to peripheral nerves, a similar process can take place in the pathways of the central nervous system (CNS), though at a much slower rate and without subsequent regeneration [5,6,7].

Central nervous system WD has been largely revealed by animal studies, and although they have provided considerable information on the individual steps of WD, they have limited relevance to human processes because of differences in temporal and spatial dynamics between species [3,8,9,10].

Since longitudinal histopathological analysis is impossible, our knowledge about the time profile of WD in the CNS after stroke is incomplete; the results of the scarce histological studies so far do not provide a clear description of the process. A comprehensive analysis has been carried out from spinal sections following a middle cerebral artery (MCA) stroke and spinal cord injury [11,12], while the remaining studies either focus only on later processes or use histological methods that are not suitable for the detection of early abnormalities [13,14]. Evidence of corticospinal tract (CST) degeneration following ischemic (ISC) or hemorrhagic (HEM) strokes has also been confirmed by several MR-based investigations, many of which provide some information about the dynamics over time, yet the exact attribution of the MR phenomena to the pathological steps of WD is not yet completely clear [15,16,17,18,19,20,21,22,23,24,25,26,27].

The relevance of our research is supported by the high prevalence of stroke patients all over the world. As WD may be related to poor functional outcome, a better understanding of the pathological processes behind it might be clinically important, promoting research on neuroprotection, neurorehabilitation, and influencing early therapeutic decisions [17,26,28,29].

Using histopathological methods to study the pyramids of the medulla oblongata, our objective was to contribute to an accurate mapping of CST degeneration following stroke. We focused on exploring the early processes that take place during the first month, and in addition, we compared the time course of WD following ISC and HEM stroke.

## 2. Patients and Methods

### 2.1. Study Population

Post-mortem investigations of the CSTs in the pyramid of the medulla oblongata were performed on autopsy samples from 22 patients. Eleven patients (HEM group) had extensive striatocapsular hypertensive cerebral hemorrhage involving the full internal capsule, while nine patients (ISC group) had a total MCA territory infarct, all proven by antemortem imaging studies and also by full post-mortem neuropathological examination (by TK). In addition, two patients with long survival times (330 and 450 days) following their ischemic stroke were also studied (Table 1), but without including their data in the statistical analysis. Patients had no history of any other CNS disorder and had no other CNS pathology at autopsy, while clinically, all had persistent severe hemiparesis or hemiplegia.

### 2.2. Methods

All methods were performed in accordance with the relevant guidelines and regulations.

After removal, the brains were fixed in a 10% formaldehyde solution. The maximum post-mortem interval between death and brain fixation was 52 h, and there was no difference between the ISC and HEM cases (44 ± 11 and 42 ± 12 h, respectively). Following confirmation of the clinical diagnosis through neuropathological examination, blocks from the midsection of the pyramid of the medulla oblongata were excised and embedded in paraffin wax. Next, 10 um thick sections were prepared and immunostained for CD68 (clone KP1, DAKO, Glostrup, Denmark; dilution 1:200) and neurofilament H (NF-H) (clone N52.1.7, Leica Biosystems, Richmond, IL, USA; dilution 1:300) using a VECTASTAIN Elite RTU kit (Vector Laboratories, Burlingame, CA, USA) and counterstained with hematoxylin (Reanal Labor, Budapest, Hungary) for nuclear staining to help morphological orientation. Klüver–Barrera (KB) stain (Luxol Fast Blue and cresyl violet; Reanal Labor, Budapest, Hungary) was used for myelin staining.

Photomicrographs were taken at 40×, 100×, and 200× magnifications with a Zeiss Axio Imager M2 type light microscope with a Zeiss Axiocam 503 colour digital camera, and image analysis was performed using the ZEN 2.6 computer software (Carl Zeiss AG, Oberkochen, Germany). We analyzed the CSTs (determined by their anatomic localization) in the pyramids of the medulla oblongata on both sides.

As the sections were prepared above the decussation, the ipsilesional side is the pathologic (lesioned) one, while the contralesional side is the intact (normal, non-lesioned) one, which was used as the control.

Cases were subdivided into four groups based on the survival times after stroke: very early (post-stroke days (D) 1–2), early (D3–7), intermediate (D10–18), and late (D21–90) groups. The definition of the groups was defined according to the appearance of pathological features, which showed consistent patterns across cases within each group.

### 2.3. Immunhistochemical Analysis

CD68 density was expressed as a ratio of CD68-positive area in μm^2^ to the total measured area in mm^2^. CD68-positive macrophages were manually segregated on the analyzed images and removed from the analysis. The measurements were performed separately in both pyramids, and the results were statistically evaluated (see below).

Because of the swelling of the axons in the acute stage, measurement of axon density in NF-H-stained sections was not possible, so the changes were evaluated by observation of the homogeneity of the staining, arrangement, and size distribution of the axons, and their spatial density.

### 2.4. Morphological Analysis of the Myelin Sheaths

KB-stained sections were evaluated by observation, at 600× magnification, and the surface of the myelin sheaths, the completeness of the myelin rings, and the proportion of the stained and non-stained areas were described.

### 2.5. Statistical Analysis

The distribution of continuous variables was checked using the Shapiro–Wilk test. For multivariate analysis, we used the general linear model (GLM). Density values between the pathological and non-pathological sides were compared using the Wilcoxon matched pairs test in the total group and separately in the ISC and HEM subgroups. Spearman’s rank-order correlation was used to evaluate the relationship between density values and time elapsed from stroke separately on the pathological and non-pathological sides. Kruskal–Wallis ANOVA was used to compare CD68 density values among the 4 groups based on the time after stroke. To identify the independent predictors of CD68 density, mixed-effects regression was used with stroke type (ISC or HEM), side of the lesion, and sex as categorical variables and time from stroke and age as continuous variables as possible predictors. The mixed-effects approach was necessary due to repeated measurements on both sides of the patients. Time from stroke was modelled non-linearly in the regression model. Models with quadratic, square root, and reciprocal relationships were investigated and evaluated visually using Akaike and Bayesian information criteria. A difference of *p* < 0.05 was considered statistically significant. TIBCO statistica v. 14.0 and R v. 4.4.1 (USA) were used for data analysis.

## 3. Results

**Figure 1:**
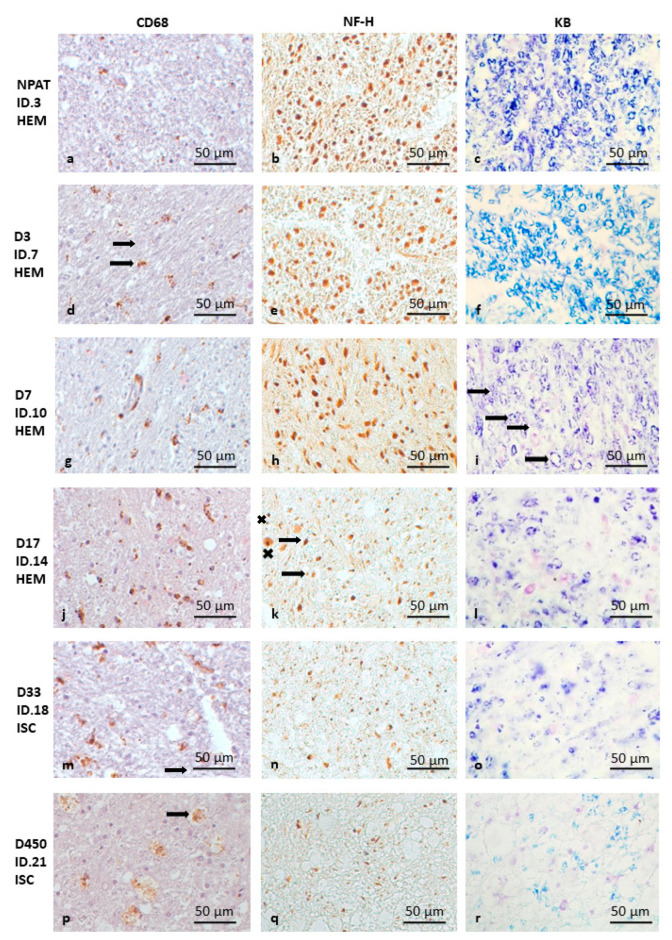
Pathology of CST degeneration in the pyramids of the medulla oblongata following HEM and ISC stroke (as the changes are similar, ISC and HEM cases are not shown separately). CD68 immunohistochemistry (**a**,**d**,**g**,**j**,**m**,**p**), NF-H immunohistochemistry (**b**,**e**,**h**,**k**,**n**,**q**), and Klüver–Barrera staining (**c**,**f**,**i**,**l**,**o**,**r**). The bar in the bottom right corner of the pictures indicates 50 μm. Intact (NPAT) side of the pyramid in the medulla oblongata (**a**–**c**) for comparison (D3 ISC). Very early HEM case (day 3—D3) with microglial activation (**d**) (brown spots) (the arrow shows ramified microglia) but without signs of myelin (**e**) or axon degeneration (**f**). Early (day 7—D7) HEM case with incipient axon and myelin destruction (arrows show breaking of the myelin rings) (**g**–**i**). Intermediate (day 17—D17) (**j**–**l**) (HEM) and late (ISC) case (day 33—D33) (**m**–**o**) with marked myelin degradation (**l**,**o**), axon degeneration (**k**,**n**) (the arrow shows a shrunken axon, while “x” shows a swollen axon) and increasing microglial activity (**j**,**m**). Very late stage of CST degeneration (D450 ISC) with several macrophages (arrow) and extensive loss of axons and myelin (**p**–**r**).

Early in the first 2 days (D1–2) after stroke, CD68 density is considerable in the CSTs on both sides of the medulla oblongata; however, the difference between the two sides is not yet apparent. The axonal structure and myelin sheaths are still intact. The microscopic anatomy is the same as on the intact, non-pathological (NPAT) side (Figure 1a–c).

In the following time interval of 3–7 days (D3–7), on the ipsilesional (pathological) side, the CD68 density increases on the third day (Figure 1d), while NF-H (Figure 1e) and myelin (Figure 1f) stainings are normal. (Figure 1g). On D7, in circumscribed areas of the ipsilesional pyramid, the axonal structure shows the first signs of disintegration on NF-H staining, with swollen axonal cross-sections (Figure 1h). In addition, early signs of myelin degradation (with breaking of some of the myelin rings) also appear (Figure 1i).

In the D10–18 time period, the CD68 density difference between the two sides is still very striking (Figure 1j). The signs of axonal degeneration are obvious; the axons are swollen and their structures lose integrity, thus destroying the regular structural pattern in the ipsilesional medulla oblongata (Figure 1k). In addition, further degradation of myelin sheaths was observed on KB-stained sections: the myelin rings lose their regular roundness and appear broken in several places (Figure 1l).

After one month (Figure 1m–o), besides the continuing strong CD68 density difference (Figure 1m) and obvious signs of ongoing axon degeneration (Figure 1n), the signs of myelin degradation become more evident, i.e., a decrease in staining intensity with only sparse visible myelin rings (Figure 1o). The severity of the pathology remains similar after three months.

In the two additional late cases (D330 and D450), the number of CD68-positive cells is still significantly increased on the pathological side (Figure 1p), with a high density of microglia, together with macrophages along the surface of the medullary pyramid. Axon density is dramatically reduced, with the remaining fibers being thinner compared to the intact side (Figure 1q). Myelin staining is significantly diminished, but signs of myelin degradation are still present (Figure 1r).

Comparing the HEM and ISC groups, the overall CD68 density on the ipsilesional side (HEM n:11 (mean ± SD): 12,515 ± 8127); (ISC n:9 (mean ± SD): 14,871 ± 5639); (all cases n:20 (mean ± SD): 13,353 ± 6898) was approximately twice that on the contralesional side (HEM n:11 (mean ± SD): 6937 ± 2151); (ISC n:9 (mean ± SD): 7866 ± 3751); (all cases n:20 (mean ± SD): 7436 ± 2898) in both patient groups (Wilcoxon matched pairs test *p* (all cases): 0.0005), and no difference was observed between HEM and ISC when comparing the pathological sides (*p*: 0.4780).

When examining the whole group, considering time as a continuous variable, CD68 density on the ipsilesional side significantly correlated with survival time (Spearman’s rank-order correlations n:20 *p*: <0.0001).

A similar result is obtained when four time points are formed; there is a significant difference in density between time groups on the ipsilesional side (Kruskal–Wallis ANOVA n:20 *p* = 0.0047). No similar correlation was observed on the contralesional side.

**Figure 2:**
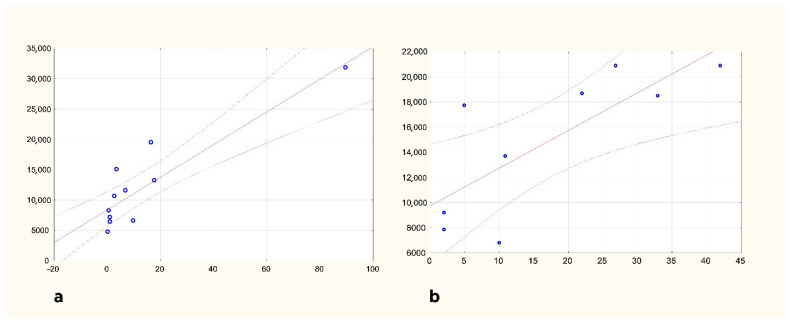
Changes in CD68 density in the ipsilesional pyramid of the medulla oblongata HEM (**a**) and ISC (**b**) groups are shown separately. Horizontal axis: survival in days; vertical axis: CD68 density in μm^2^/mm^2^.

Analyzing HEM and ISC separately, density on the ipsilesional side is correlated with time elapsed in both patient groups, but no such correlation on the contralesional side could be proven (Spearman’s rank-order correlations HEM n:11 *p*: 0.005; ISC n:9 *p*: 0.013). Through multivariate analysis using a mixed-effects regression model, independent predictors of CD68 density were survival time (*p* = 0.003 for the linear term and *p* = 0.248 for the non-linear term) and side of lesion (ipsi- vs. contralesional; *p* < 0.001) but not sex, age, or stroke type. The relationship between CD68 and survival time density was modelled using a reciprocal curve, based on the visual assessment and information criteria. CD68 density still increases after 3 months of survival (Figure 2). Based on the long-survival time cases, microglial activation is still present 1.5 years after the stroke

## 4. Discussion

Previous data have shown that microglial activation in the CST of the cervical spinal cord (hereafter the decussation, i.e., on the contralateral side) is evident on days 3–4 after injury, but earlier cases were not histologically analyzed [30,31,32]. In our very early cases (D1–2), although microglia presence is observed in the pyramidal tracts on both sides in the first 2 days, there is no difference between the sides in this aspect. The first sign of CST degeneration at the level of the medulla is visible from day 3, when the density of microglia is clearly and statistically higher on the ipsilesional, i.e., pathological side. Our previous studies have shown a similar pattern in the cervical spinal cord, suggesting that the activation of the microglia along the course of the CST is simultaneous and might be independent of the proposed rostro-caudal spreading of the WD [15,31,33].

After one week, there is clear microglial activation on the ipsilesional side, while myelin degradation starts, and there are signs of axonal damage on NF-H staining. This is earlier than in previous observations, and most of the MR-based studies have not demonstrated a clear MR abnormality in this period [11,12,17,25,26,30,33].

In the intermediate group (D10–18), signs of axon degeneration are evident on the ipsilesional side, and myelin loss is also observed. In a previous comprehensive myelin-focused study, investigating myelin integrity using immunohistochemistry targeting several different proteins, MAG (myelin-associated glycoprotein) immunohistochemistry also showed myelin involvement early, at day 14, while immunohistochemistry of the other proteins indicated that myelin was still intact [12,34]. In our cases, structural damage to the myelin was already seen at this time, although this observation is weakened by the unquantified, observational assessment of KB-stained sections. Nevertheless, it is possible that slight damage to the myelin structure already causes staining deficiency with easier stain washout (which is not seen on the contralesional, normal side), but degradation of most myelin-associated proteins has not yet begun [34,35,36]. Other studies of human CNS WD using conventional myelin staining methods are available only from a later period [13,14]. Based on the spatial and temporal correlation of pathological data and the results of previous MR studies [8,9,11,12,17,25,37,38,39], axon degeneration and myelin damage may be the combined cause of MR abnormalities consistent with WD from the first week onwards. The advanced axon degeneration seen from week 3 onwards and the axon and myelin loss in the following year have been confirmed by numerous previous studies [11,12,13,14,32,40].

Although our present study did not focus on the late stages of CST degeneration following stroke (cases with 330 and 450 D survival), the persistent microglial and macrophage activity merits further studies to define the maladaptive or neuroprotective role of their activation. The use of CD68 as a marker of microglia is based on its better sensitivity to detect activated microglia in the subcortical white matter compared to iba-1 [41], while being aware of the limitation that it also stains the macrophages, which were differentiated based on their different morphology.

In the present study, we found no differences in either microglial activation or in the dynamics of axon and myelin degradation between the HEM and ISC disease groups. Thus, the finding that the CST degenerates at a similar rate after HEM as ISC may argue in favor of early surgical intervention in hemorrhagic stroke, i.e., early decompression of brain tissue through the removal of the hematoma may be beneficial [40,42,43,44,45] and may prevent WD. However, as we do not have samples from all time points and individual differences may exist, some differences cannot be excluded, and it would be interesting to investigate these in a larger group of patients.

## 5. Conclusions

No differences in the dynamics of WD were found between HEM and ISC stroke during the first month after the insult. At the level of the ipsilesional medulla oblongata, microglial activation is initiated on day 3, followed by axon degeneration and concomitant myelin degradation after day 7, a process that is further enhanced during the first month.

## Figures and Tables

**Table 1:** Study population. Twenty patients (1–20) were used for the statistical analysis, while patients 21 and 22 were used to illustrate the long-term features of WD. Survival is in hours (H) or days (D).

Patient ID	Sex, Age	Type of Stroke	Side of the Lesion	Survival
1	M78	HEM	R	5H
2	M67	HEM	L	22H
3	M75	HEM	R	29H
4	F78	HEM	R	30H
5	F86	ISC	L	2D
6	F80	ISC	R	2D
7	M66	HEM	L	3D
8	M79	HEM	R	4D
9	F90	ISC	R	5D
10	M77	HEM	R	7D
11	M65	HEM	R	10D
12	F82	ISC	R	10D
13	F83	ISC	L	11D
14	F73	HEM	L	17D
15	M64	HEM	L	18D
16	F90	ISC	R	22D
17	M76	ISC	R	27D
18	F60	ISC	R	33D
19	M59	ISC	R	42D
20	M52	HEM	R	90D
21	M62	ISC	L	450D
22	M69	ISC	L	330D

## Data Availability

The data presented in this study are available from the corresponding author due to legal reasons upon request.

## Abbreviations

CNS	central nervous system
CST	corticospinal tract
GLM	general linear model
HEM	Hemorrhagic
ISC	Ischemic
KB	Klüver–Barrera
MCA	middle cerebral artery
NF-H	neurofilament H
WD	Wallerian degeneration
